# Host RhoA Signaling Controls Filamentous vs. Spherical Morphogenesis and Cell-to-Cell Spread of RSV via Lipid Raft Localization: Host-Directed Antiviral Target

**DOI:** 10.3390/microorganisms13071599

**Published:** 2025-07-07

**Authors:** Manoj K. Pastey, Lewis H. McCurdy, Barney S. Graham

**Affiliations:** 1 Department of Veterinary Biomedical Sciences, Oregon State University, Corvallis, OR 97330, USA; 2 Department of Infectious Diseases, Wake Forest University School of Medicine, Charlotte, NC 28204, USA; lewis.mccurdy@atriumhealth.org; 3 Vaccine Research Center, National Institute of Allergy and Infectious Diseases, National Institutes of Health, Bethesda, MD 20892, USA; bgraham@nih.gov

**Keywords:** respiratory syncytial virus, Rhosin, RhoA, lipid raft

## Abstract

*Respiratory syncytial virus* (RSV) is a major human respiratory pathogen, particularly affecting infants, the elderly, and immunocompromised individuals. RSV exists in both spherical and filamentous forms, with the filamentous morphology associated with enhanced infectivity and cell-to-cell spread. Here, we demonstrate that RhoA, a small GTPase involved in cytoskeletal regulation, is essential for filamentous RSV morphogenesis through its role in organizing lipid raft microdomains. Rhosin, a selective RhoA inhibitor developed through structure-guided screening, disrupts GEF–RhoA interactions to block RhoA activation. The pharmacological inhibition of RhoA with Rhosin significantly reduced filamentous virion formation, disrupted RSV fusion (F) protein colocalization with lipid rafts, and diminished cell-to-cell fusion, without affecting overall viral replication. Scanning electron microscopy revealed that Rhosin-treated infected HEp-2 cells exhibited fewer and shorter filamentous projections compared to the extensive filament formation seen in untreated cells. β-galactosidase-based fusion assays confirmed that reduced filamentation corresponded with decreased cell-to-cell fusion. The biophysical separation of RSV spherical and filamentous particles by sucrose gradient velocity sedimentation, coupled with fluorescence and transmission electron microscopy, showed that Rhosin treatment shifted virion morphology toward spherical forms. This suggests that RhoA activity is critical for filamentous virion assembly, which may enhance viral spread. Immunofluorescence microscopy using lipid raft-selective dyes (DiIC_16_) and fusion protein-specific antibodies revealed the strong co-localization of RSV proteins with lipid rafts. Importantly, the pharmacological inhibition of RhoA with Rhosin disrupted F protein partitioning into raft domains, underscoring the requirement for intact lipid rafts in assembly. These findings highlight a novel role for host RhoA signaling in regulating viral assembly through raft microdomain organization, offering a potential target for host-directed antiviral intervention aimed at altering RSV structural phenotypes and limiting pathogenesis.

## 1. Introduction

*Respiratory syncytial virus* (RSV) is a leading cause of severe lower respiratory tract infections in infants, the elderly, and immunocompromised individuals [1]. Despite decades of research, effective antiviral therapies remain limited, highlighting the urgent need to elucidate key mechanisms underlying RSV pathogenesis. Emerging evidence points to virus–host cytoskeletal interactions as critical drivers of RSV assembly, morphogenesis, and intercellular spread [2].

RSV actively manipulates host cytoskeletal components—namely actin filaments, microtubules, and intermediate filaments—to support filamentous virion formation and efficient viral transmission [3,4,5]. The filamentous morphology of RSV particles has been associated with increased infectivity and enhanced syncytium formation, facilitating direct cell-to-cell dissemination [6]. Notably, RSV activates the ARP2/3 complex to promote actin polymerization and filopodia formation in infected A549 cells, enhancing viral budding and spread [7]. RSV also disrupts F-actin structures and reduces the actin-binding protein cortactin, compromising epithelial barrier function [8].

Key regulators of cytoskeletal remodeling include the Rho family of small GTPases—RhoA, Rac1, and Cdc42—which orchestrate actin dynamics, cell morphology, and membrane organization [9]. RhoA, in particular, is activated in RSV-infected cells and localizes within lipid rafts—cholesterol- and sphingolipid-rich membrane microdomains that facilitate signal transduction and cytoskeletal regulation [10]. The disruption of lipid rafts perturbs RhoA activation and downstream cytoskeletal functions. Additionally, RSV exploits Rab11-dependent microtubule and actin-mediated trafficking via Rab11 family interacting proteins (Rab11-FIPs), enhancing intracellular viral transport [11].

**Figure 1:**
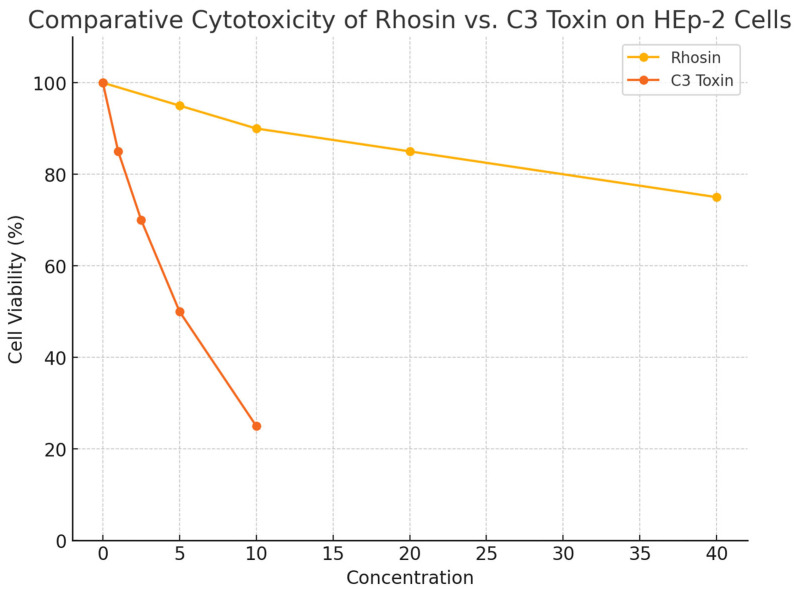
**Comparative cytotoxicity profiles of Rhosin and C3 toxin on HEp-2 cells.** MTT assay-based cytotoxicity curves of HEp-2 cells treated with Rhosin (0, 5, 10, 20, 40 µM) or C3 toxin (0, 1, 2.5, 5, 10 µg/mL) for 24 h. Data are shown as mean ± standard deviation (SD) of percentage viability relative to untreated controls, from three independent experiments.

Previous studies using C3 toxin—a broad-spectrum Rho inhibitor—highlighted the role of RhoA in filamentous virion formation and syncytium induction [10]. However, the C3 toxin’s clinical utility is hampered by nonspecific cytotoxicity, poor cell permeability, and pan-Rho inhibition that complicates data interpretation and reduces clinical applicability. In contrast, our study used Rhosin, a small-molecule RhoA-specific inhibitor developed through in silico virtual screening and structure-based design, specifically targeting the GEF–RhoA interaction site [12]. It exhibits enhanced selectivity, cellular uptake, and reduced cytotoxicity, making it a promising tool for dissecting RhoA-specific pathways and improving therapeutic potential, making it a more suitable candidate for potential clinical applications [12]. Consistent with this, our MTT-based cytotoxicity assay (Figure 1) confirmed that Rhosin maintains high cell viability compared to C3 toxin, validating its use for exploring host–virus interactions and as a potential therapeutic approach.

Building on Gower et al.’s findings, we employed Rhosin to selectively inhibit RhoA and investigate its role in RSV-induced morphological and functional changes. Our data demonstrate that RhoA inhibition does not significantly impair viral replication (as determined by PFU counts) but markedly alters virion morphology and cell fusion dynamics. Scanning electron microscopy revealed that Rhosin-treated cells exhibited a marked reduction in filamentous virions compared to the large aggregates of long filaments typically observed in untreated cells. Complementary analyses using sucrose gradient velocity sedimentation, fluorescence microscopy, and transmission electron microscopy further confirmed that RhoA inhibition shifts the balance from filamentous to spherical RSV particles. Immunofluorescence studies using the raft-selective dye DiIC_16_(3) and anti-F protein antibodies revealed that Rhosin disrupts the association of RSV fusion proteins with lipid rafts, implicating RhoA in raft-dependent virion assembly.

Together, these findings implicate RhoA as a critical host factor for filamentous virion formation, syncytium development, and efficient RSV dissemination. Targeting host signaling pathways that modulate viral morphology—rather than replication alone—offers a novel therapeutic avenue to attenuate RSV spread. This study contributes to our understanding of virus–host interactions and identifies RhoA as a promising target for anti-RSV strategies.

## 2. Materials and Methods

### 2.1. Virus Propagation and Cell Culture

The A2 strain of RSV was obtained from the American Type Culture Collection, Manassas, VA, USA (ATCC; VR-1540). RSV A2 stocks were propagated in HEp-2 cells, and viral titers were determined by plaque-forming unit (PFU) assays [13] to ensure consistent infection doses across experiments.

HEp-2 cells (ATCC; CCL-23) and HEK-293T cells (ATCC; CRL-3216) were cultured in Dulbecco’s Modified Eagle Medium (DMEM) supplemented with 10% fetal bovine serum and 1% penicillin-streptomycin at 37 °C in a humidified 5% CO_2_ atmosphere.

### 2.2. Comparative Cytotoxicity of Rhosin and C3 Toxin

Cell viability in response to Rhosin treatment (20 µM) was assessed using trypan blue exclusion and MTT assays at 12, 24, and 48 h post-treatment. No significant cytotoxicity was observed at this concentration.

Given the known limitations of the C3 toxin in terms of cytotoxicity and cell permeability, we undertook a comparative analysis of Rhosin, a specific RhoA inhibitor, and C3 toxin cytotoxicity in HEp-2 cells to establish a safer and more specific approach for RhoA inhibition in RSV research. Briefly, HEp-2 cells were seeded at 1 × 10^4^ cells per well in 96-well plates and were allowed to adhere overnight. To evaluate cytotoxicity, the cells were treated with increasing concentrations of Rhosin (0, 5, 10, 20, and 40 µM; Sigma-Aldrich, Saint Louis, MO, USA) or C3 toxin (0, 1, 2.5, 5, and 10 µg/mL; Cytoskeleton Inc., Denver, CO, USA) for 24, 48, and 72 h. DMSO and PBS served as vehicle controls for Rhosin and the C3 toxin, respectively. Following treatment, cell viability was assessed using an MTT assay by adding MTT reagent (0.5 mg/mL) for 3 h at 37 °C. After incubation, the media were removed, and the resulting formazan crystals were solubilized in DMSO. Absorbance was measured at 570 nm using a microplate reader, and cell viability was expressed as a percentage relative to untreated controls. All treatments were performed in triplicate, and the data were presented as the mean ± standard deviation.

### 2.3. RSV Replication Kinetics in Rhosin-Treated Cells

To assess the reproducibility and temporal impact of Rhosin treatment on RSV replication, HEp-2 cells were infected with RSV at a multiplicity of infection (MOI) of 1 and treated with 20 µM Rhosin. Supernatants were collected at 24, 48, and 72 h post-infection, and viral titers were determined using plaque-forming unit (PFU) assays as previously described ([13,14], and Section 2.4). Untreated infected cells served as controls. Each condition was performed in triplicate, and the data were analyzed using two-way ANOVA to compare viral titers across treatment groups and time points.

### 2.4. Effect of Rhosin Treatment on RSV Replication and Plaque Formation in HEp-2 Cells

For infection studies, HEp-2 cells were seeded at appropriate densities and infected with RSV at a MOI of 1 for 1 h. To investigate the role of RhoA, the cells were treated with Rhosin at 20 µM concentrations determined by preliminary dose–response studies. Rhosin was added to the culture medium prior to infection and maintained throughout the infection period (72 h). Control groups were treated with vehicle alone. Viral replication was assessed using a standard PFU assay [13]. Briefly, after a defined incubation period post-infection, the supernatants were collected and serially diluted. Diluted samples were overlaid onto approximately 1 × 10^5^ HEp-2 cell monolayers and incubated for 1 h before the agarose overlay was applied. After 72 h, plates were fixed with methanol, and RSV-specific immunoperoxidase staining was performed [14]. RSV plaques were counted in each well to quantify infectious virions, comparing Rhosin-treated samples with untreated controls.

### 2.5. Scanning Electron Microscopy (SEM)

To examine virion morphology, HEp-2 cells on 12 mm coverslips were pre-treated for 16 h with 20 µM Rhosin or mock-treated and then infected with RSV (MOI 1), with treatments maintained post-adsorption. Controls included uninfected untreated cells. At 48 h post-infection, the cells were fixed in 4% glutaraldehyde (1 h), post-fixed with 1% osmium tetroxide (15 min), dehydrated through graded ethanol washes, critical point dried, sputter coated with gold, and imaged using Hitachi S4200 SEM, Pleasanton, CA, USA. SEM images were captured to visualize the differences in filamentous and spherical virion forms between Rhosin-treated and untreated cells.

### 2.6. Sucrose Gradient Velocity Sedimentation

Sucrose gradient sedimentation was employed to separate RSV particles based on density and shape. HEp-2 cells in 25-cm^2^ flasks were either left untreated or treated with 20 µM Rhosin for 24 h and then infected with RSV (MOI of 0.5). After a 72 h infection, the infected cells were harvested and layered over a continuous 15 to 60% sucrose gradient. The virus was then separated by velocity centrifugation at 14,000 rpm for 10 min at 4 °C in a Sorvall Sure Spin 630 centrifuge, Sorvall Thermo Scientific, Waltham, MA, USA. Two-milliliter fractions were collected, and plaque assays were performed with the fractions as described previously [13]. Fractions were also analyzed by fluorescence microscopy and transmission electron microscopy (TEM) to distinguish between spherical and filamentous virions, thereby quantifying the impact of RhoA inhibition on particle morphology.

### 2.7. Fluorescence Microscopy and Transmission Electron Microscopy (TEM)

Fluorescence microscopy was used to assess the distribution and localization of RSV proteins in infected cells. Specimens for fluorescence microscopy were prepared on coverslips and stained. Briefly, 50 µL of RSV-containing supernatant from infected cells was smeared on a coverslip and allowed to air dry. The virus particles were fixed with 3.7% formaldehyde in PBS for 30 min. The samples were washed twice with PBS-Tween 20 and blocked with 5% nonfat dry milk in PBS for 30 min. The cells were stained with a 1:500 dilution of anti-RSV antibody (Maine Biotechnology Services, Portland, ME, USA) prepared in 1% nonfat dry milk and incubated for 1 h at room temperature. This was followed by staining with a 1:1000 dilution of goat anti-mouse IgG (H + L) secondary antibody conjugated to Rhodamine (ThermoFisher Scientific, Waltham, MA, USA), also for 1 h at room temperature. After three washes with PBS-Tween 20, the coverslips were mounted on microscope slides. The specimens were viewed with a Zeiss AxioPlan 2 fluorescence microscope, Carl Zeiss Microscopy, Dublin, CA, USA and pictures were taken with a Hamamatsu ORCA-ER digital camera, Hamamatsu Corporation, Bridgewater, NJ, USA.

Additionally, TEM provided high-resolution images of virion ultrastructure. For evaluations of particles separated by velocity sedimentation, the bands associated with the peak fractions were concentrated by centrifugation onto a 60% sucrose cushion. A sample from the concentrated band was applied to a grid, stained with 0.5% uranyl acetate, and examined using a Hitachi H-7000 transmission electron microscope, Hitachi High-TechAmerica, Inc., Schaumburg, IL, USA, allowing for detailed visualization of RSV morphology in both Rhosin-treated and untreated conditions.

### 2.8. Beta-Galactosidase Cell-to-Cell Fusion Assay

The functional impact of altered virion morphology on cell-to-cell fusion was measured using a beta-galactosidase reporter assay [15]. Briefly, HEp-2 cells were divided into two populations: **A. Effector cells** were infected with vaccinia virus vTF7-3 (MOI = 10) to express T7 polymerase, and then transfected with plasmids for RSV glycoproteins F, G, SH, and M (under T7 promoter control). At 5 h post-transfection, the cells were trypsinized, resuspended at 2 × 10^7^ cells/mL in MEM containing 2.5% FBS, and 20 μg/mL of either the Rhosin or the control media, or left untreated, incubated overnight at 32 °C, and later adjusted to 1 × 10^6^ cells/mL in Opti-MEM. **B. Reporter cells were** infected with a recombinant vaccinia virus engineered to express β-galactosidase under the T7 promoter (provided by E.A. Berger, National Institutes of Health, Bethesda, MD, USA). At 5 h post-infection, these cells were trypsinized and suspended in Opti-MEM at a concentration of 1 × 10^6^ cells/mL.

Equal volumes (100 μL) of the effector and reporter cell suspensions were mixed in triplicate wells in a 96-well tissue culture plate and incubated at 37 °C for 4 h to allow for cell-to-cell fusion. Two complementary approaches were used to measure fusion events: **1**. **Colorimetric Lysate Assay:** β-galactosidase activity was quantified using the CPRG (chlorophenol red-β-D-galactopyranoside) assay kit (G-Biosciences, Saint Louis, MO, USA, Cat. #786-651), which provides a sensitive colorimetric readout at 570–595 nm. Infected or transfected HEp-2 cells were lysed with Mammalian Cell PE LB™ buffer (G-Biosciences, Saint Louis, MO, USA, Cat. #786-180), freeze-thawed, and clarified by centrifugation. In total, 20 µL of the clarified lysate was added to a 96-well plate and incubated with 130 µL of 1× CPRG substrate at 37 °C. Reactions were stopped after color development using the provided stop solution, and absorbance was read in triplicate using a microplate reader. The readings were normalized to mock-transfected controls and the protein concentration of lysates. β-galactosidase activity was calculated based on a chlorophenol red standard curve and expressed as ng of product per well. **2**. **In Situ X-Gal Staining:** Fixed cells were stained with an X-Gal solution, and blue syncytia were visualized under phase-contrast microscopy. This dual-assay approach allowed for both quantitative measurement and visual confirmation of RSV F-induced cell-to-cell fusion and facilitated the assessment of the inhibitory effects of the Rhosin on fusion events.

### 2.9. Synthetic RSV Protein Expression and Transfection

Briefly, protein sequences corresponding to the RSV A2 strain structural proteins fusion (F), attachment glycoprotein (G), small hydrophobic protein (SH), and matrix (M), as documented in GenBank: KT992094.1 [16], were reverse-translated into human cell-preferred codons using the Wisconsin Genetics Computer Group (GCG) software package version 10.3. Subsequently, oligonucleotides covering these four genes were synthesized commercially by Sigma-Genosys, with each oligonucleotide being 75 bases long with an overlap of 25 bases between consecutive segments. The codon-optimized genes were then assembled into expression vectors derived from the pNGVL-3 plasmid backbone and verified through sequencing to confirm accuracy and integrity. Plasmid DNA was purified by double cesium chloride gradient ultracentrifugation to ensure high purity suitable for transfection. The cells were transfected with 2 µg each of the plasmids encoding the F, G, SH, and M proteins (totaling 8 µg DNA per transfection) using the calcium phosphate precipitation method described by Chen and Okayama [17].

### 2.10. Immunofluorescence and Lipid Raft Labeling

To investigate the association between RSV structural proteins and lipid raft microdomains, we employed immunofluorescence microscopy coupled with selective lipid raft labeling. DiIC_16_(3) (1,1-dihexadecyl-3,3,3,3-tetramethylindocarbocyanine perchlorate) consists of a long acyl chain, which allows for its incorporation into the more rigid, ordered lipid domains, compared to DiIC_12_(3) (1,1-didodecyl-3,3,3,3-tetramethylindocarbocyanine perchlorate), which labels the fluid non-raft membrane components [18]. Briefly, HEK-293T cells were seeded on coverslips and transfected with 2 µg each of the plasmids encoding the F, G, SH, and M proteins (totaling 8 µg DNA per transfection) using the calcium phosphate precipitation method described by Chen and Okayama [17]. The vector backbone without viral inserts was used as filler DNA to maintain consistent total DNA amounts across experimental conditions. At 48 h post-transfection, cells seeded on coverslips were chilled on ice and incubated with a 1:100 dilution of lipid raft-selective dye DiIC_16_(3) or non-raft-selective dye DiIC_12_(3) dissolved in ethanol for 15 min. Subsequently, the cells were fixed with 3.7% formaldehyde for 10 min at room temperature, permeabilized with 0.5% Triton X-100 in phosphate-buffered saline (PBS) for 10 min and blocked with 5% nonfat milk in PBS for 15 min to minimize nonspecific binding. The cells were then incubated for 1 h at room temperature with primary antibodies: mouse anti-RSV F monoclonal antibody (Millipore Sigma, Burlington, MA, USA), diluted at 1:500 in 3% milk-PBS. Following washing with PBS-Tween-20, the cells were incubated for 1 h with Alexa Fluor 488-conjugated secondary antibodies (anti-mouse IgG, Molecular Probes, Eugene, OR, USA) diluted to 1:1000 in 3% milk–PBS. Images were captured using a Zeiss Axioplan fluorescence microscope, Carl Zeiss Microscopy, Dublin, CA, USA, equipped with a 100× oil immersion objective, and digital images were acquired using an Axiocam camera, Carl Zeiss Microscopy, Dublin, CA, USA.

### 2.11. Rhosin-Mediated Disruption of RSV Protein Assembly in Lipid Rafts

To determine the effect of RhoA inhibition in RSV structural protein assembly in lipid rafts, we used Rhosin to inhibit RhoA in HEK-293T cells. HEK-293T cells were seeded on coverslips and were either left untreated or treated with 20 µM Rhosin for 24 h and then transfected with pNGVL-3 plasmids encoding the F, G, SH, and M proteins. Treatment with 20 µM Rhosin was continued post transfection until 48 h. At 48 h post-transfection, the cells were washed twice with PBS and then labeled with dyes and antibodies as described above. The samples were mounted and imaged by fluorescence microscopy. Rhosin treatment resulted in the disruption of F protein colocalization with DiIC_16_ (3), supporting the role of cholesterol-rich microdomains in viral particle assembly.

### 2.12. Data Analysis

All experiments were conducted in triplicate. The data were analyzed using appropriate statistical methods, including *t*-tests or ANOVA, to compare differences between Rhosin-treated and control groups. Significance was set at *p* < 0.05. Data presentation included the means ± standard error of the mean (SEM), with graphical representation generated using GraphPad Prism, Version 10.5.0 or standard software packages.

Overall, these methods enabled a comprehensive evaluation of the role of RhoA in RSV infection, specifically addressing its impact on virion morphology, syncytium formation, and the efficiency of viral spread.

## 3. Results

### 3.1. Rhosin Exhibits Lower Cytotoxicity than C3 Toxin in HEp-2 Cells

To compare the cytotoxic profiles of Rhosin and C3 toxin, HEp-2 cells were treated with increasing concentrations of each compound, and cell viability was measured using an MTT assay. As shown in Figure 1 and Table 1, Rhosin exhibited minimal cytotoxicity, maintaining cell viability above 75% even at 40 µM, aligning with previous reports on its favorable cellular safety profile [12]. In contrast, C3 toxin showed significantly higher cytotoxicity, with cell viability dropping to 50% at 5 µg/mL and further to 25% at 10 µg/mL. These findings underscore the methodological and therapeutic advantage of using Rhosin as a RhoA-specific inhibitor with lower toxicity and better cellular permeability, providing a more suitable and interpretable tool for studying RSV–host cytoskeletal interactions and for potential clinical translation.

### 3.2. Rhosin Does Not Affect RSV Replication Kinetics over Time

**Figure 2:**
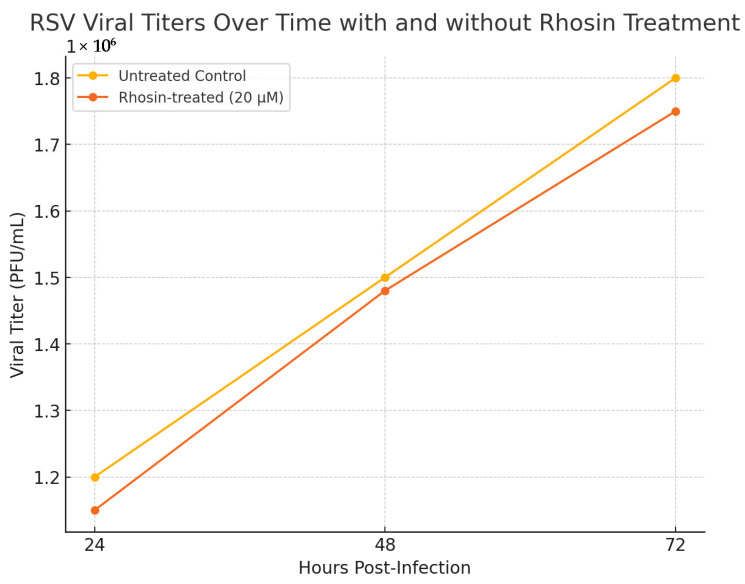
**RSV viral titers over time in untreated and Rhosin-treated HEp-2 cells**. HEp-2 cells were infected with RSV at an MOI of 1 and treated with 20 µM Rhosin. Viral titers in culture supernatants were measured by plaque-forming unit (PFU) assays at 24, 48, and 72 h post-infection. *Y*-axis represents plaque counts per well at different treatment conditions. Data represent the mean ± standard deviation of three independent experiments.

To further assess the reproducibility and temporal impact of Rhosin treatment on RSV replication, we measured RSV titers in supernatants collected at 24, 48, and 72 h post-infection from HEp-2 cells treated with 20 µM Rhosin or vehicle control. As shown in Figure 2, statistical analysis using two-way ANOVA revealed no significant differences in viral titers between the Rhosin-treated and control groups at any of the tested time points (*p* > 0.05). These results confirm that Rhosin treatment does not interfere with RSV replication kinetics over time, reinforcing that the observed effects on virion morphology and cell-to-cell spread are independent of viral genome replication or particle production. This consistency across multiple time points and independent experiments strengthens the conclusion that RhoA inhibition by Rhosin selectively modulates RSV particle morphology and fusion dynamics without compromising viral replication.

### 3.3. RhoA Inhibition Alters RSV Virion Morphology

**Figure 3:**
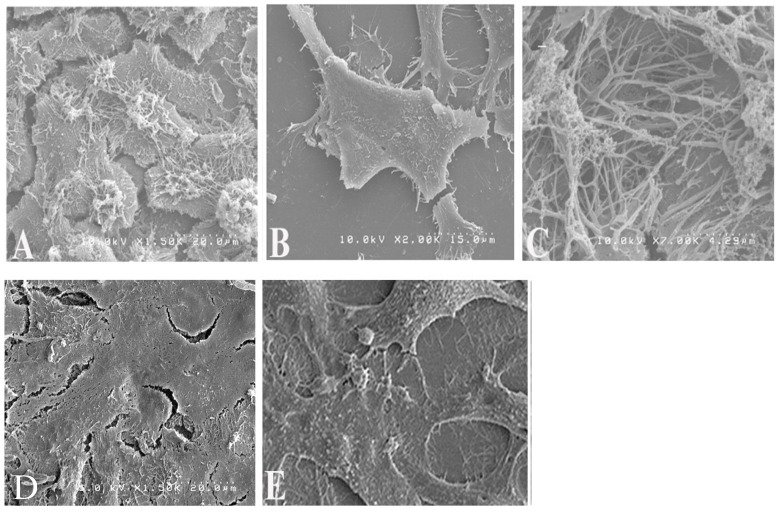
**Scanning electron microscopy images showing the impact of Rhosin on RSV particle morphology.** (**A**,**C**) Untreated RSV-infected HEp-2 cells showing prominent filamentous virions on the cell surface. (**B**) Rhosin-treated (20 µM) RSV-infected cells exhibiting reduced filamentous structures and more blunted filaments at 48 h post-infection. Controls shown are uninfected HEp-2 cells (**D**) and uninfected and Rhosin-treated HEp-2 cells (**E**). Magnification ×6300.

To assess the role of RhoA in RSV particle morphogenesis, HEp-2 cells were treated with Rhosin and infected with RSV. As shown in Figure 3, scanning electron microscopy (SEM) revealed that untreated RSV-infected HEp-2 cells displayed abundant long filamentous virions, forming large aggregates on the cell surface (Figure 3A,C). In contrast, Rhosin-treated cells showed a significant reduction in filamentous virions, with an increased presence of spherical viral particles (Figure 3B). These findings suggest that active RhoA signaling is critical for filamentous RSV formation.

### 3.4. Sucrose Gradient Sedimentation Confirms Morphological Shift

**Figure 4:**
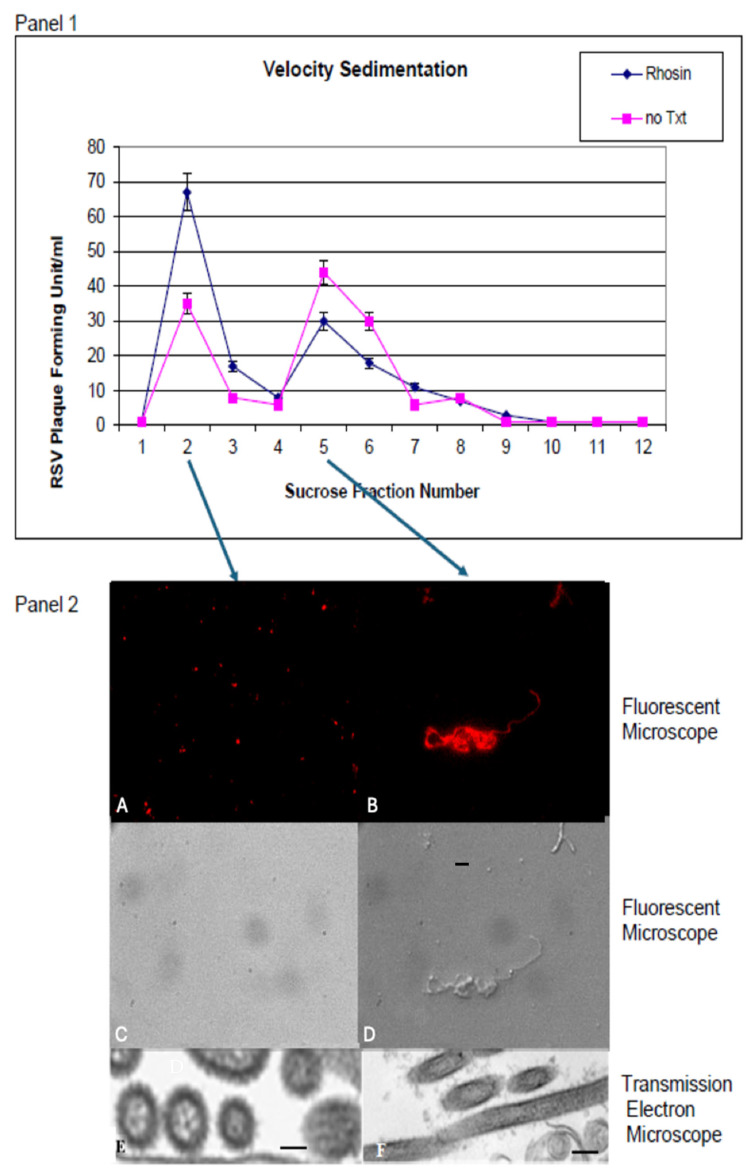
**Separation of spherical and filamentous RSV particles following sucrose gradient sedimentation. Panel 1** shows the distribution of infectious RSV particles across sucrose gradient fractions, comparing untreated and Rhosin-treated samples. **Panel 2** presents representative fluorescence microscopy (**A**–**D**; magnification ×40) and transmission electron microscopy images (**E**,**F**; Bar = 100 nm), illustrating the morphological characteristics of virions from peaks corresponding to spherical (fraction #2) and filamentous (fraction #5) particles. Images (**A**,**B**) show immunofluorescent staining of RSV F protein using anti-F monoclonal antibody followed by Rhodamine-conjugated goat anti-mouse secondary antibody. Images (**C**,**D**) present corresponding bright-field images of the same fields to visualize cell morphology.

To further quantify changes in virion morphology, RSV particles were separated by velocity sedimentation on a 15–60% sucrose gradient. As shown in Figure 4, RSV particles from untreated cells showed a distribution characteristic of both filamentous and spherical virions, with a greater proportion of filamentous forms in the heavier fractions (Fraction 5). In contrast, Rhosin treatment resulted in a distinct shift, with a higher proportion of spherical particles accumulating in the lighter fractions (Fraction 2). This confirms that RhoA inhibition promotes a transition from filamentous to spherical virion morphology. TEM and the fluorescence microscopy of gradient fractions confirmed that fraction 2 contained predominantly spherical particles, while fraction 5 was enriched with filaments, validating the shift in morphology induced by RhoA inhibition (Figure 4).

### 3.5. Plaque Assays Show No Significant Impact of Rhosin on Viral Replication

**Figure 5:**
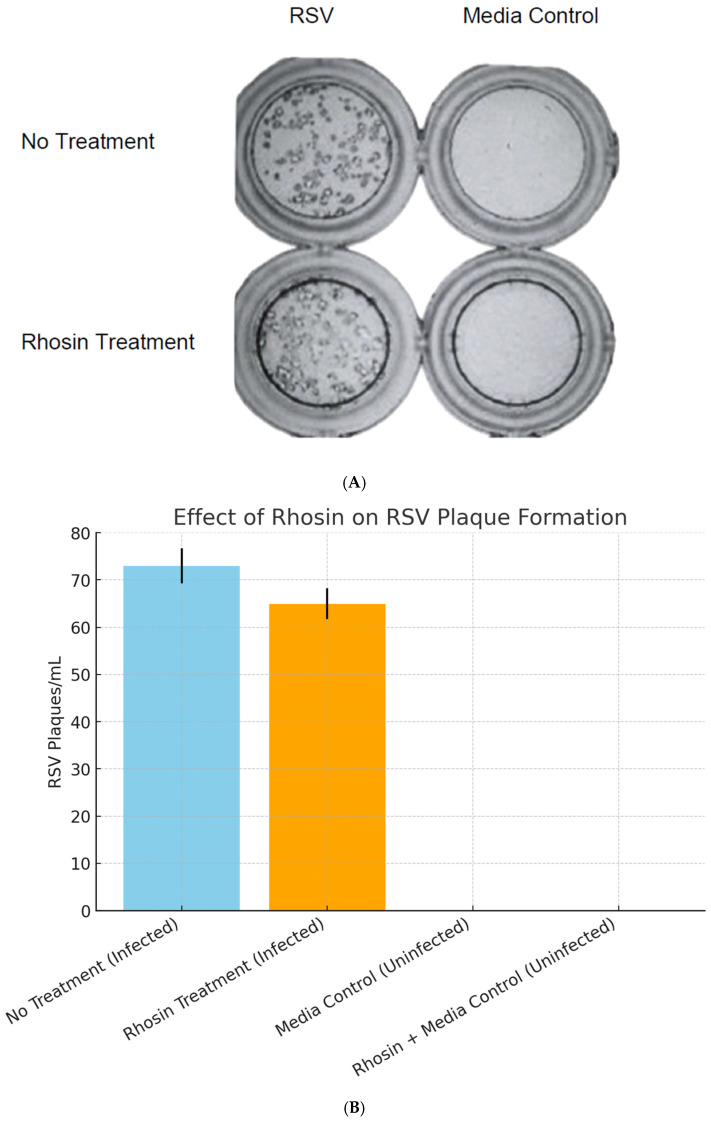
(**A**). **RSV replication in the presence or absence of Rhosin treatment.** Plaque assay results showing RSV replication at 72 h post-infection in HEp-2 cells with and without 20 µM Rhosin treatment. Control groups with media alone, with or without Rhosin, showed no detectable plaques. The figure represents one of the three independent experiments conducted. (**B**). **Effect of Rhosin treatment on RSV plaque formation in HEp-2 cells.** Bar graph showing RSV plaque-forming units (PFU/mL) in HEp-2 cells infected with RSV at an MOI of 1 and treated with 20 µM Rhosin or left untreated. Culture supernatants were collected at 72 h post-infection. Media control and Rhosin-treated media control groups remained negative for RSV plaques. Error bars represent 5% of the mean PFU/mL, and data shown are representative of one of three independent experiments.

To determine whether Rhosin treatment impacts RSV replication, we quantified RSV plaque formation in supernatants collected from HEp-2 cells infected with RSV (72 h post-infection at an MOI of 1) and treated with or without Rhosin. Despite the morphological changes induced by Rhosin treatment, plaque-forming unit (PFU) assays demonstrated that viral replication remained unaffected (Figure 5A,B). Viral titers from Rhosin-treated and untreated cells were comparable with no statistically significant difference, indicating that RhoA inhibition does not impair the ability of RSV to replicate (Figure 5A,B). This suggests that RhoA is primarily involved in virion assembly, fusion dynamics, and morphology rather than genome replication or viral protein production.

### 3.6. RhoA Inhibition Reduces RSV-Induced Cell-to-Cell Fusion

**Figure 6:**
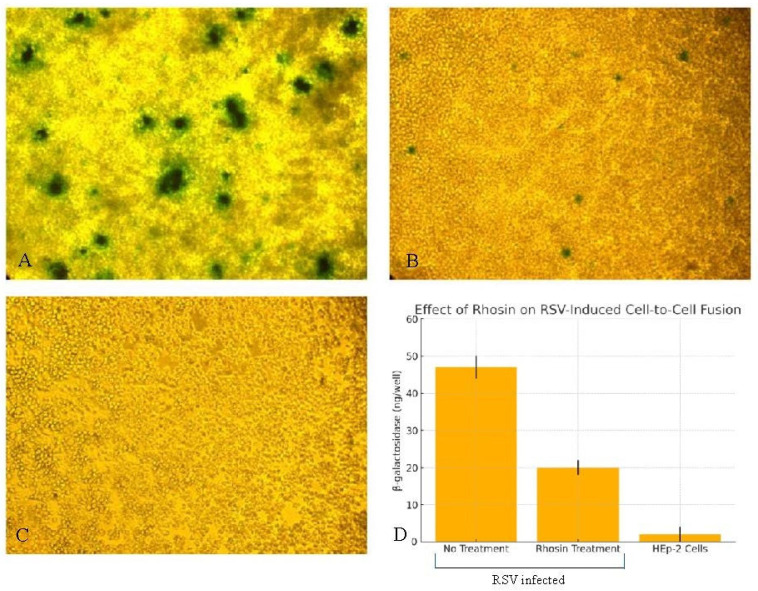
**Effect of Rhosin on RSV-induced cell-to-cell fusion measured by β-galactosidase activity.** HEp-2 cells expressing RSV F, G, and SH glycoproteins were untreated (**A**) or treated with Rhosin (**B**). HEp-2 cells not expressing RSV envelope proteins were treated with Rhosin (**C**). The above-mentioned cells were then mixed with another cell population infected with recombinant vaccinia virus expressing β-galactosidase, then assessed by in situ X-gal staining (**A**–**C**); magnification, ×10) or quantitative colorimetric lysate assay (**D**). Syncytia were counted in 10 high-power fields (*n* = 3 wells per group). (**D)**: Equal volumes of cell lysates and 2× substrate solution were mixed, and the rates of substrate hydrolysis were monitored by measuring absorbance at 590 nm with a spectrophotometer. The data are presented as β-galactosidase (ng/well) produced by the cells. The data represent one representative experiment of three independent replicates. Error bars indicate standard deviation.

To assess whether the altered virion morphology influenced viral spread, we used a β-galactosidase-based fusion assay to quantify cell-to-cell fusion. Syncytia were counted in 10 high-power fields (*n* = 3 wells per group). As shown in Figure 6, untreated cells expressing RSV glycoproteins induced robust syncytium formation (29.8 ± 1.2), reflected in high β-galactosidase activity and large multinucleated cells by X-Gal staining. However, Rhosin-treated cells exhibited significantly reduced fusion (*p* < 0.01), with fewer and smaller syncytia (8.3 ± 1.4), as evidenced by a lower β-galactosidase signal and fewer multinucleated syncytia in X-Gal staining assays. These findings suggest that filamentous RSV plays a crucial role in promoting direct cell-to-cell fusion, and disrupting RhoA signaling reduces viral dissemination through this mechanism.

### 3.7. RSV Proteins Preferentially Localize to Lipid Rafts, Disrupted by Rhosin

**Figure 7:**
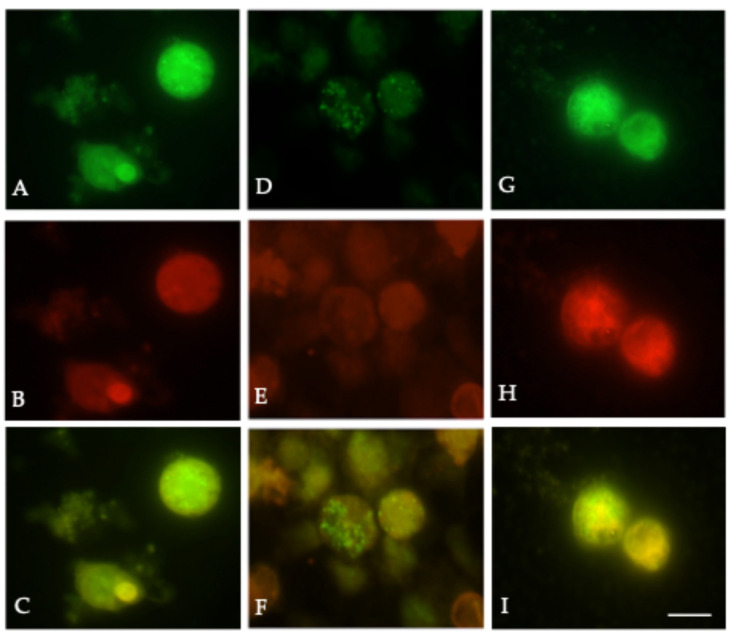
**RhoA inhibition disrupts RSV fusion protein localization to lipid raft microdomains.** (**A**–**C**) HEK-293T cells transfected with plasmids encoding RSV F, G, SH, and M proteins using the calcium phosphate precipitation method were labeled with the raft-selective dye DiIC_16_(3) and stained with anti-RSV F monoclonal antibody followed by Alexa Fluor 488-conjugated secondary antibody. Strong co-localization of RSV F protein (green) with DiIC_16_(3)-labeled lipid rafts (red) was observed in merged images (yellow). (**D**–**F**) Parallel transfections were labeled with the non-raft-selective dye DiIC_12_(3). Minimal co-localization between RSV F protein and DiIC_12_(3)-labeled membranes was observed, indicating selective raft association of RSV proteins. (**G**–**I**) Cells treated with 20 µM Rhosin showed disrupted co-localization between RSV F protein and DiIC_16_(3), with distinct separation of green (F protein) and red (lipid raft) signals in merged images, indicating that RhoA activity is required for fusion protein incorporation into lipid rafts. Images were captured using fluorescence microscopy with a 100× oil immersion objective. Pearson’s correlation analysis confirmed significantly higher co-localization between RSV F and DiIC_16_ (r = 0.82) versus DiIC_12_ (r = 0.14; *p* < 0.0001). Rhosin treatment reduced F–raft co-localization, supporting the role of RhoA in RSV assembly at raft microdomains. Scale bar: 10 µm.

**Figure 8:**
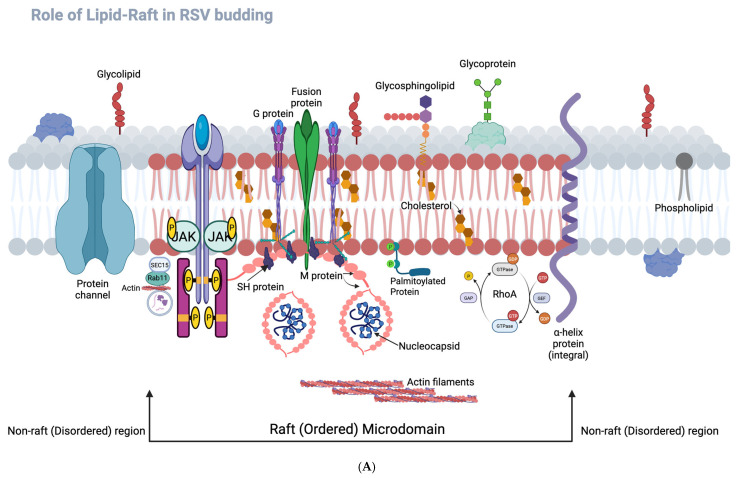

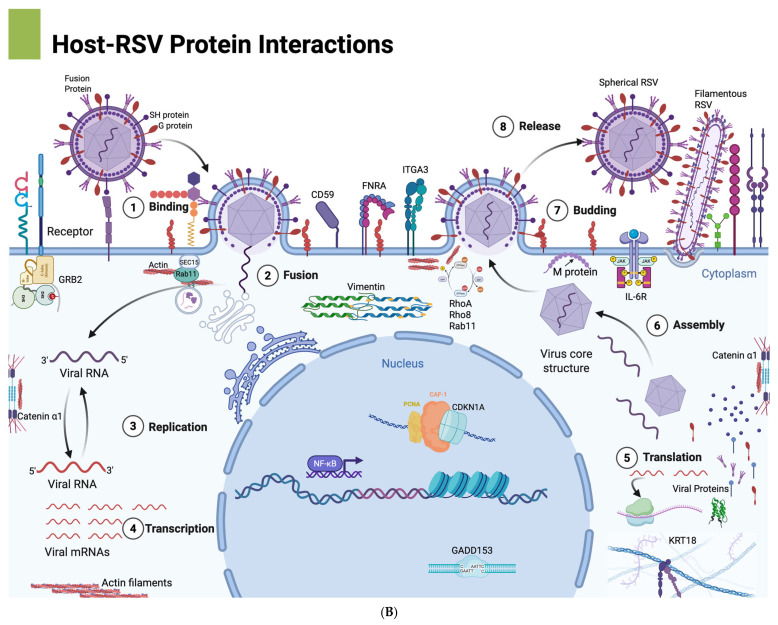
(**A**). **Schematic representation of RSV protein assembly within lipid raft and non-raft membrane domains.** The diagram illustrates the differential localization of RSV structural proteins within the host cell plasma membrane. Lipid rafts (depicted as cholesterol- and sphingolipid-enriched microdomains) serve as ordered membrane regions that facilitate the assembly of RSV fusion (F), attachment (G), and matrix (M) proteins. These proteins concentrate within raft domains, promoting efficient virion assembly and filamentous morphogenesis. In contrast, non-raft regions (fluid, disordered lipid environments) do not support organized RSV protein assembly. The disruption of RhoA signaling impairs F protein targeting of lipid rafts, leading to defective filament formation and reduced cell-to-cell fusion. This schematic highlights the functional role of raft domains in spatially organizing viral components during the RSV life cycle. Diagram created with BioRender.com. (**B**). **Schematic representation of the RSV life cycle and host–virus interactions.** This diagram illustrates key stages of the *Respiratory syncytial virus* (RSV) life cycle, highlighting both spherical and filamentous virion morphologies and the host cellular components involved. RSV attachment and entry (1) occur via surface glycoproteins (F, G, SH), followed by fusion (2) with the host membrane. The intracellular trafficking of the viral ribonucleoprotein (RNP) complex is facilitated by cytoskeletal elements, including actin filaments and vimentin. Viral replication (3) and transcription (4) occur in inclusion bodies, after which newly synthesized viral proteins (5) and genomes are transported to the plasma membrane. Assembly (6) and budding (7) preferentially occur at lipid raft microdomains, which are enriched with cholesterol and sphingolipids. Notably, lipid rafts facilitate efficient filamentous virion assembly, although spherical particles may also utilize raft components to a lesser extent. Release of progeny virions (8) occurs from the plasma membrane. Diagram created with BioRender.com.

With immunofluorescent markers that selectively partition into liquid-ordered and fluid domains of the membranes, we sought to determine the colocalization of viral proteins and cellular proteins with lipid microdomains. Although all envelope glycoproteins were transfected using the calcium phosphate precipitation method described by Chen and Okayama [17], only F was visualized by immunofluorescence to focus on fusion-competent localization. At 48 h post-transfection, HEK-293T cells expressing RSV F, G, SH, and M proteins were labeled with either lipid raft-selective dye DiIC_16_(3) or non-raft dye DiIC_12_(3), for 15 min on ice. After fixation, cells were labeled with a mouse anti-RSV F monoclonal antibody (1:500 dilution), followed by Alexa Fluor 488-conjugated anti-mouse IgG (1:1000 dilution). As shown in Figure 7A–C, the RSV F protein colocalized strongly with DiIC_16_(3)-labeled domains, while little overlap was observed with DiIC_12_(3) (Figure 7D–F). Pearson correlation analysis confirmed the significantly higher co-localization of F with raft markers (r = 0.82) compared to non-raft dyes (r = 0.14; *p* < 0.0001), supporting the hypothesis that RSV preferentially assembles at cholesterol-rich lipid rafts. To interrogate the functional relevance of RhoA and the co-localization of RSV proteins to lipid rafts, we inhibited RhoA using Rhosin. Rhosin treatment disrupted the co-localization of the fusion protein with DiIC_16_(3)-labeled lipid raft-like microdomains (Figure 7G–I), indicating that RhoA activity supports the proper localization of RSV structural proteins within cholesterol-rich microdomains essential for filamentous assembly. These findings indicate that the correct localization of F protein to lipid rafts, mediated by RhoA, is required for efficient membrane fusion and cell-to-cell spread (Figure 6). A schematic of RSV protein localization to lipid rafts is shown in Figure 8A.

### 3.8. Graphical Representation of RSV Life Cycle and Host–Raft Interactions

To provide an integrated overview of RSV replication and morphogenesis, we generated schematic diagrams (Figure 8A,B) summarizing virus–host interactions and the role of RhoA-mediated lipid raft organization.

Figure 8A presents a graphical model of RSV protein localization to lipid rafts and the assembly process. It highlights how host-derived lipid raft microdomains serve as critical platforms for viral glycoprotein concentration, membrane curvature, and budding. Notably, lipid rafts facilitate efficient filamentous virion assembly, although spherical particles may also utilize raft components to a lesser extent.

Figure 8B extends this model to depict the complete RSV lifecycle, including attachment, entry, transcription, replication, protein trafficking, and virion assembly. It contrasts the morphogenetic pathways of filamentous versus spherical particles and emphasizes the dependence of filamentous morphogenesis on intact RhoA signaling and lipid raft integrity. The disruption of this pathway by Rhosin treatment results in altered viral morphology, reduced membrane fusion, and impaired cell-to-cell spread.

The figure also shows several host genes that are significantly upregulated during RSV infection (≥2-fold, *p* < 0.01), including those involved in apoptosis (e.g., MCL1, CASP4), endoplasmic reticulum (ER) stress response (GADD153, CDKN1A/p21), cytoskeletal remodeling (VIM, TUBA1), immune signaling (IL6), and cell adhesion (FNRA) (our unpublished observations). Notably, several of these upregulated host proteins—vimentin (VIM), RhoA, fibronectin receptor α (FNRA), and CD59—are known to associate with lipid raft microdomains, suggesting a coordinated role in supporting RSV assembly and morphogenesis at raft-enriched membrane domains. The observed increase in IL-6 further reflects the proinflammatory environment elicited during RSV infection. Together, these host responses underscore the intricate interplay between viral assembly and host cytoskeletal, membrane, and signaling pathways.

Together, these schematics reinforce the mechanistic findings of this study and provide a conceptual framework linking host cytoskeletal regulation with RSV assembly and dissemination.

The figure also shows host genes significantly upregulated in RSV-infected cells (≥2-fold, *p* < 0.01), involved in apoptosis (MCL1, CASP4), ER stress response (GADD153, CDKN1A [p21]), cytoskeletal reorganization (VIM, TUBA1), immune signaling (IL6), and cell adhesion (FNRA) (our unpublished observations). Several of these host proteins—VIM (vimentin), RHOA, FNRA (fibronectin receptor α), and CD59—are associated with lipid raft microdomains, suggesting a coordinated role in viral assembly and morphogenesis. Upregulated IL-6 illustrates the inflammatory response component of RSV infection. This integrative schematic underscores the interplay between RSV and host cytoskeletal, membrane, and signaling machinery during infection. The diagram was created with BioRender.com.

## 4. Discussion

This study highlights the critical role of RhoA signaling in regulating respiratory syncytial virus morphology, syncytium formation, and cell-to-cell fusion, and further establishes lipid rafts as critical platforms for RSV assembly and release. While RSV exists in both filamentous and spherical forms, filamentous particles are considered more effective at mediating direct intercellular spread [10,19]. MTT viability assays confirmed that Rhosin exhibits significantly lower cytotoxicity than C3 toxin (Figure 1, Table 1). Rhosin displayed minimal cytotoxic effects, maintaining high cell viability across the tested concentrations, while the C3 toxin showed pronounced, dose-dependent toxicity. These findings underscore Rhosin’s improved safety profile and specificity, supporting its application as a more suitable RhoA inhibitor for investigating RSV–host cytoskeletal interactions, and as a promising candidate for therapeutic intervention.

To further assess the reproducibility and temporal impact of Rhosin treatment on RSV replication, viral titers were measured at 24, 48, and 72 h post-infection in HEp-2 cells treated with 20 µM Rhosin or left untreated. Consistent with the viral titer analysis across multiple time points, our findings confirm that Rhosin-mediated RhoA inhibition does not impact RSV replication kinetics, underscoring that its primary effects are restricted to altering virion morphology and reducing cell-to-cell spread.

While RhoA inhibition with Rhosin did not significantly impact RSV replication, it profoundly altered virion structure by shifting RSV particles from filamentous to spherical forms, as corroborated by scanning electron microscopy (SEM), sucrose gradient velocity sedimentation, fluorescence microscopy, and transmission electron microscopy (TEM), which collectively confirmed that RhoA inhibition disrupts the formation of filamentous RSV particles (Figure 3, Figure 4 and Figure 5). These findings suggest that RhoA signaling plays a pivotal role in RSV assembly and dissemination. Our data corroborates previously published results showing that blocking RhoA signaling with C3 toxin did not affect the RSV replication but affected filament formation [10]. However, the C3 toxin has other toxic effects on host cells, and C3 is not ideal for clinical use due to its low cellular penetration. These results reinforce that RhoA signaling is indispensable for filamentous RSV assembly, a phenotype associated with enhanced cell-to-cell fusion and dissemination.

Filamentous RSV particles are thought to facilitate viral spread through enhanced cell-to-cell fusion and syncytium formation, thereby bypassing the need for extracellular release and re-entry [19]. Filamentous morphology may allow for continuous virion transmission through intercellular junctions, bypassing immune surveillance and enhancing syncytium-mediated spread, which has been linked to increased disease severity in vivo [19]. The functional consequences of disrupted filament formation were demonstrated using a β-galactosidase fusion assay, which showed a significant reduction in syncytium formation in Rhosin-treated cells (Figure 6), correlating with the loss of filamentous virions observed by SEM (Figure 3). This supports the notion that filamentous RSV is more efficient in mediating direct cell-to-cell transmission, a key factor in viral pathogenesis. The disruption of filament formation may limit RSV’s ability to spread efficiently within the host, potentially offering a novel antiviral strategy.

Despite these morphological and functional changes, plaque assays confirmed that RSV replication remained unaffected by RhoA inhibition between Rhosin-treated and control groups (Figure 5A,B). This suggests that RhoA signaling is not essential for viral genome replication or particle production but is instead crucial for virion assembly and dissemination. Sucrose gradient velocity sedimentation further validated this, showing a distinct shift toward spherical RSV particles upon RhoA inhibition by Rhosin treatment (Figure 4).

Our lipid raft localization studies demonstrated that the RSV F protein preferentially associates with raft-selective domains, and this association is disrupted by RhoA inhibition (Figure 7), underscoring the requirement for intact lipid rafts in assembly. Lipid rafts are dynamic, cholesterol- and sphingolipid-enriched microdomains of the cell membranes that act as platforms for signal transduction, protein sorting, and pathogen entry and facilitate membrane curvature and scission; their disruption impairs filamentous virion formation and viral dissemination. These results are consistent with the literature showing that many enveloped viruses, including HIV, influenza, coronavirus, Ebola, and measles, hijack lipid rafts for their assembly and budding [20,21,22,23,24,25]. These domains concentrate viral proteins, facilitate glycoprotein clustering, and interact with host factors that coordinate membrane curvature and scission. As such, lipid rafts have emerged as strategic targets for antiviral drug development [26,27].

Our findings align with previous studies indicating that host cytoskeletal dynamics play a crucial role in RSV infection [2]. The dependence of filamentous RSV on RhoA activity highlights a potential vulnerability that could be exploited therapeutically. RhoA exerts its effects through several downstream effectors, including Rhokinase/ROCK/ROK and mDia1, which are known to regulate actin cytoskeleton organization and stress fiber formation [28,29]. These pathways likely contribute to the structural support necessary for filamentous RSV morphogenesis. Additionally, RhoA-mediated activation of cofilin and LIM kinase pathways may influence membrane curvature and fusion protein trafficking [30]. Targeting host factors like RhoA or its downstream effectors, rather than viral components, may reduce the likelihood of resistance development while limiting viral spread. By shifting the balance from filamentous to spherical virion formation, such interventions may reduce the virus’s ability to form syncytia and spread efficiently, thereby limiting disease progression even in the absence of a direct antiviral effect on replication. This approach could be particularly valuable in controlling RSV infections in high-risk populations, such as infants and immunocompromised individuals [1].

The conceptual frameworks illustrated in Figure 8A,B further contextualize our findings. Figure 8A highlights the spatial organization of RSV assembly at lipid raft versus non-raft membrane domains, illustrating how RSV structural proteins—particularly F, G, and M—preferentially assemble within cholesterol-rich lipid rafts to promote filamentous morphogenesis. The disruption of this organization via RhoA inhibition impairs proper membrane targeting of the F protein, likely contributing to the observed shift toward spherical virion morphology and reduced cell-to-cell fusion. Complementarily, Figure 8B presents an overview of the RSV life cycle, integrating host cellular components that facilitate or respond to infection. It visually maps key stages including viral entry, replication, and assembly, and highlights host cytoskeletal elements (e.g., actin filaments, vimentin), inflammatory mediators (e.g., IL-6), and upregulated genes with roles in apoptosis, stress response, and membrane organization. Several of these—VIM, RHOA, FNRA, and CD59—are known to localize to lipid rafts, suggesting a coordinated host–virus interface at these membrane microdomains (our unpublished observations). Together, these schematics support a model in which RSV exploits RhoA-dependent lipid raft assembly and host cytoskeletal networks to drive filamentous virion formation and efficient spread. Although both spherical and filamentous particles utilize lipid raft components during assembly, filamentous virions exhibit a greater dependence on these microdomains.

In conclusion, our findings identify RhoA signaling as a critical regulator of RSV filamentous morphogenesis and cell-to-cell spread. RhoA coordinates the localization of RSV fusion (F) proteins to lipid raft microdomains, enabling efficient assembly of filamentous virions and promoting syncytium formation. The pharmacological inhibition of RhoA using Rhosin alters virion morphology, disrupts F protein partitioning into lipid rafts, and significantly reduces syncytium formation—all without affecting total viral replication. These results suggest that RhoA, or its downstream effectors, may represent promising targets for host-directed antiviral intervention.

However, the broader implications of RhoA inhibition on viral infectivity and transmission dynamics remain to be established, particularly in physiologically relevant in vivo models. Future studies should evaluate the impact of RhoA inhibition on RSV pathogenesis and explore potential side effects of targeting this pathway in host tissues. Additionally, identifying specific downstream effectors of RhoA involved in filament formation may yield novel antiviral strategies that selectively impair RSV assembly and spread.

## Figures and Tables

**Table 1:** Dose-dependent cytotoxicity of Rhosin and C3 toxin on HEp-2 cells.

Concentration (Rhosin, µM)	Viability Rhosin (%)	Concentration (C3 Toxin, µg/mL)	Viability C3 Toxin (%)
0	100	0	100
5	95	1	85
10	90	2.5	70
20	85	5	50
40	75	10	25

HEp-2 cells were exposed to increasing concentrations of Rhosin (0–40 µM) or C3 toxin (0–10 µg/mL) for 24 h. Cell viability was determined using the MTT assay and is presented as percentage viability relative to untreated control cells (100%). Values represent the mean from three independent experiments.

## Data Availability

The original contributions presented in this study are included in the article. Further inquiries can be directed to the corresponding author.

## Abbreviations

RSV: Respiratory Syncytial Virus; RhoA: Ras Homolog Family Member A; MOI: Multiplicity of Infection; TEM: Transmission Electron Microscopy; SEM: Scanning Electron Microscopy; CPRG: Chlorophenol red-β-D-galactopyranoside; DiIC_16_ (3): 1,1-dihexadecyl-3,3,3,3-tetramethylindocarbocyanine perchlorate; DiIC_12_ (3): 1,1-didodecyl-3,3,3,3-tetramethylindocarbocyanine perchlorate.

## References

[1] Hall C.B., Weinberg G.A., Iwane M.K., Blumkin A.K., Edwards K.M., Staat M.A., Auinger P., Griffin M.R., Poehling K.A., Erdman D. (2009). The burden of respiratory syncytial virus infection in young children. N. Engl. J. Med..

[2] Collins P.L., Graham B.S. (2008). Viral and host factors in human respiratory syncytial virus pathogenesis. J. Virol..

[3] Jeffree C.E., Brown G., Aitken J., Su-Yin D.Y., Tan B.H., Sugrue R.J. (2007). Ultrastructural analysis of the interaction between F-actin and respiratory syncytial virus during virus assembly. Virology.

[4] Kallewaard N.L., Bowen A.L., Crowe J.E. (2005). Cooperativity of actin and microtubule elements during replication of respiratory syncytial virus. Virology.

[5] Hu M., Bogoyevitch M.A., Jans D.A. (2020). Impact of Respiratory Syncytial Virus Infection on Host Functions: Implications for Antiviral Strategies. Physiol. Rev..

[6] Liljeroos L., Krzyzaniak M.A., Helenius A., Butcher S.J. (2013). Architecture of respiratory syncytial virus revealed by electron cryotomography. Proc. Natl. Acad. Sci. USA.

[7] Paluck A., Osan J., Hollingsworth L., Talukdar S.N., Saegh A.A., Mehedi M. (2021). Role of ARP2/3 Complex-Driven Actin Polymerization in RSV Infection. Pathogens.

[8] Gao N., Raduka A., Rezaee F. (2022). Respiratory syncytial virus disrupts the airway epithelial barrier by decreasing cortactin and destabilizing F-actin. J. Cell Sci..

[9] Ridley A.J. (2006). Rho GTPases and actin dynamics in membrane protrusions and vesicle trafficking. Trends Cell Biol..

[10] Gower T.L., Pastey M.K., Peeples M.E., Collins P.L., McCurdy L.H., Hart T.K., Guth A., Johnson T.R., Graham B.S. (2005). RhoA signaling is required for respiratory syncytial virus-induced syncytium formation and filamentous virion morphology. J. Virol..

[11] Cosentino G., Marougka K., Desquesnes A., Welti N., Sitterlin D., Gault E., Rameix-Welti M.-A. (2022). Respiratory syncytial virus ribonucleoproteins hijack microtubule Rab11 dependent transport for intracellular trafficking. PLoS Pathog..

[12] Shang X., Marchioni F., Sipes N., Evelyn C.R., Jerabek-Willemsen M., Duhr S., Seibel W., Wortman M., Zheng Y. (2012). Rational design of small molecule inhibitors targeting RhoA subfamily Rho GTPases. Chem. Biol..

[13] Graham B.S., Perkins M.D., Wright P.F., Karzon D.T. (1988). Primary respiratory syncytial virus infection in mice. J. Med. Virol..

[14] Murphy B.R., Sotnikov A., Paradiso P.R., Hildreth S.W., Jenson A.B., Baggs R.B., Lawrence L., Zubak J.J., Chanock R.M., Beeler J.A. (1989). Immunization of cotton rats with the fusion (F) and large (G) glycoproteins of respiratory syncytial virus (RSV) protects against RSV challenge without potentiating RSV disease. Vaccine.

[15] Pastey M.K., Samal S.K. (1997). Analysis of bovine respiratory syncytial virus envelope glycoproteins in cell fusion. J. Gen. Virol..

[16] Collins P.L., Hill M.G., Camargo E., Grosfeld H., Chanock R.M., Murphy B.R. (1995). Production of infectious human respiratory syncytial virus from cloned cDNA confirms an essential role for the transcription elongation factor from the 5′ proximal open reading frame of the M2 mRNA in gene expression and provides a capability for vaccine development. Proc. Natl. Acad. Sci. USA.

[17] Chen C., Okayama H. (1987). High-efficiency transformation of mammalian cells by plasmid DNA. Mol. Cell. Biol..

[18] Spink C.H., Yeager M.D., Feigenson G.W. (1990). Partitioning behavior of indocarbocyanine probes between coexisting gel and fluid phases in model membranes. Biochim. Biophys. Acta (BBA)—Biomembr..

[19] Leroy H., Han M., Woottum M., Bracq L., Bouchet J., Xie M., Benichou S. (2020). Virus-Mediated Cell-Cell Fusion. Int. J. Mol. Sci..

[20] Liao Z., Cimakasky L.M., Hampton R., Nguyen D.H., Hildreth J.E.K. (2001). Lipid rafts and HIV pathogenesis: Host membrane cholesterol is required for infection by HIV type 1. AIDS Res. Hum. Retroviruses.

[21] Manie S.N., Debreyne S., Vincent S., Gerlier D. (2000). Measles virus structural components are enriched into lipid raft microdomains: A potential cellular location for virus assembly. J. Virol..

[22] Scheiffele P., Rietveld A., Wilk T., Simons K. (1999). Influenza viruses select ordered lipid domains during budding from the plasma membrane. J. Biol. Chem..

[23] Nguyen D.H., Hildreth J.E.K. (2000). Evidence for budding of human immunodeficiency virus type 1 selectively from glycolipid-enriched membrane lipid rafts. J. Virol..

[24] Simons K., Ikonen E. (1997). Functional rafts in cell membranes. Nature.

[25] Bavari S., Bosio C.M., Wiegand E., Ruthel G., Will A.B., Geisbert T.W., Hevey M., Schmaljohn C., Schmaljohn A., Aman M.J. (2002). Lipid raft microdomains: A gateway for compartmentalized trafficking of Ebola and Marburg viruses. J. Exp. Med..

[26] El Khoury M., Naim H.Y. (2024). Lipid rafts disruption by statins negatively impacts the interaction between SARS-CoV-2 S1 subunit and ACE2 in intestinal epithelial cells. Front. Microbiol..

[27] Sorice M., Misasi R., Riitano G., Manganelli V., Martellucci S., Longo A., Garofalo T., Mattei V. (2021). Targeting Lipid Rafts as a Strategy Against Coronavirus. Front. Cell Dev. Biol..

[28] Kimura K., Ito M., Amano M., Chihara K., Fukata Y., Nakafuku M., Yamamori B., Feng J., Nakano T., Okawa K. (1996). Regulation of myosin phosphatase by Rho and Rho-associated kinase (Rho-kinase). Science.

[29] Watanabe N., Kato T., Fujita A., Ishizaki T., Narumiya S. (1999). Cooperation between mDia1 and ROCK in Rho-induced actin reorganization. Nat. Cell Biol..

[30] Arber S., Barbayannis F.A., Hanser H., Schneider C., Stanyon C.A., Bernard O., Caroni P. (1998). Regulation of actin dynamics through phosphorylation of cofilin by LIM-kinase. Nature.

